# Post traumatic stress symptoms and heart rate variability in Bihar flood survivors following yoga: a randomized controlled study

**DOI:** 10.1186/1471-244X-10-18

**Published:** 2010-03-02

**Authors:** Shirley Telles, Nilkamal Singh, Meesha Joshi, Acharya Balkrishna

**Affiliations:** 1 Department of Yoga Research, Patanjali Yogpeeth, Delhi-Haridwar Highway Haridwar 249402, India

## Abstract

**Background:**

An earlier study showed that a week of yoga practice was useful in stress management after a natural calamity. Due to heavy rain and a rift on the banks of the Kosi river, in the state of Bihar in north India, there were floods with loss of life and property. A week of yoga practice was given to the survivors a month after the event and the effect was assessed.

**Methods:**

Twenty-two volunteers (group average age ± S.D, 31.5 ± 7.5 years; all of them were males) were randomly assigned to two groups, yoga and a non-yoga wait-list control group. The yoga group practiced yoga for an hour daily while the control group continued with their routine activities. Both groups' heart rate variability, breath rate, and four symptoms of emotional distress using visual analog scales, were assessed on the first and eighth day of the program.

**Results:**

There was a significant decrease in sadness in the yoga group (p < 0.05, paired t-test, post data compared to pre) and an increase in anxiety in the control group (p < 0.05, paired t-test, post data compared to pre).

**Conclusions:**

A week of yoga can reduce feelings of sadness and possibly prevent an increase in anxiety in flood survivors a month after the calamity.

**Trial Registration:**

Clinical Trials Registry of India: CTRI/2009/091/000285

## Background

In August 2008 due to preceding heavy monsoon rains there was a breach in the embankments of the Kosi River near the Indo-Nepal border [1]. The breach caused loss of life and property in the north Indian state of Bihar, affecting more than 2.5 million lives.

A month after the event most of the survivors were still housed in temporary shelters as the waters had not receded. As the population consisted mainly of farmers, the possibility of them continuing their occupation appeared uncertain with the possibility of land remaining waterlogged or sand cast after the water receded.

At this stage, a month later, an attempt was made to introduce yoga as a stress-reducing strategy. The ancient Indian science of yoga includes the practice of specific postures (*asanas*), cleansing practices (*kriyas*), voluntarily regulated breathing (*pranayamas*) and meditation (*dhyana*) [2]. Hence yoga could be considered an intervention introduced fairly early after the traumatic event. Following certain traumas (e.g., sexual assault), early intervention is considered critical as the level of distress immediately after the assault has a strong positive correlation with the development of future pathologies and PTSD [3]. High distress levels at the time of assault significantly predicted increased levels of fear and anxiety in the following months [3]. The authors suggested that since the level of distress is strongly correlated to PTSD symptoms, an attempt to decrease distress immediately following the event may result in a more positive treatment outcome. However not all interventions can be considered useful soon after a trauma. For example, trauma debriefing in the initial period was found to possibly increase the risk of PTSD symptoms and certainly did not prevent the onset of PTSD [4]. Hence treatments should be continuously evaluated and modified.

Among yoga practices, *Sudarshan Kriya *yoga (SKY) is a technique which involves rhythmic hyperventilation at different rates of breathing [5]. Forty-five consenting untreated patients with melancholic depression were randomized as three treatment groups (viz., SKY, electroconvulsive therapy and imipramine). After three weeks the SKY and imipramine groups had similar scores on Beck Depression Inventory and the Hamilton Rating Scale for depression. However the SKY group had higher scores than the ECT group at three weeks. Despite this, the results suggest a possible antidepressant effect of SKY.

Apart from the yoga intervention which included SKY, another yoga program which was useful for depression and anxiety is the *Siddha Samadhi *Yoga program, in which meditation is associated with yoga breathing (*pranayama*) [6]. There were 22 volunteers with complaints of anxiety who were assigned to two groups, viz., yoga (n = 14) and a wait-list control group (n = 8). After a month of yoga practice, the yoga group had lower scores on anxiety, depression, and tension, and increased scores for well-being compared to the control group.

In the studies cited above, all the yoga programs included yoga voluntary breath regulation (*pranayama*). A review article described breathing as fundamental for physical well-being as yoga breathing 'can rapidly bring the mind to the present moment and reduce stress' [7].

Previously, a week of yoga practice which included loosening exercises, physical postures, voluntarily regulated breathing, and yoga based guided relaxation, was helpful for tsunami survivors in the Andaman islands, an archipelago in the Bay of Bengal [8]. The yoga intervention was given a month after the December 2004 tsunami. Following yoga there was a significant decrease in self-rated fear, anxiety, sadness, disturbed sleep, and in the breath rate. The main limitation of the study was that there was no control group. Comparisons were made between mainland settlers and people endogenous to the islands, where both categories of people had received yoga. Also, though there were recordings of the heart rate, breath rate, and skin resistance level, there was no objective measure to assess autonomic nervous system function, which is known to be associated with PTSD [9].

The present study was designed to assess the effect of one week of yoga practice on survivors of floods in the Indian state of Bihar, a month after the floods, with three main differences compared to the study cited above. These were: (i) a control group, since participants were randomized as yoga and wait-list control groups, (ii) recording of heart rate variability as an objective measure of autonomic nervous system function, and (iii) using a yoga program which had similar components as the program used earlier [8], though there was a greater emphasis on yoga breathing in the program used in the present study.

## Methods

### Participants

**Figure 1:**
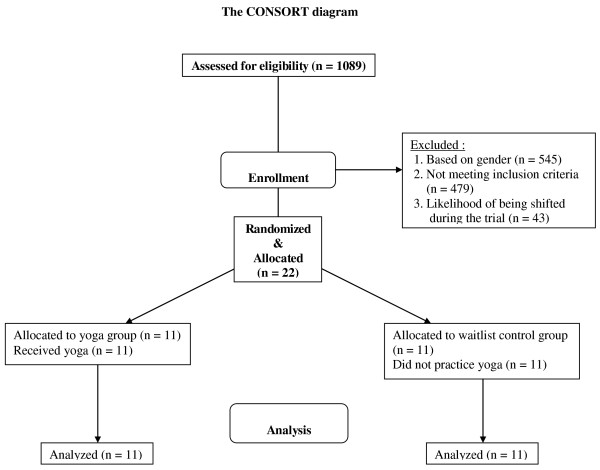
**Consort diagram summarizing participant numbers and timing of randomization, assignment, interventions and assessments**.

The participants were 1089 persons affected by the floods who were staying in a temporarily constructed camp in Bihar, India. While recruiting subjects for the study the main emphasis was on direct trauma exposure because trauma exposure is known to determine the development of PTSD [10]. The study was restricted to males as the heart rate variability is known to differ between the sexes, especially for the age group who formed the present sample [11]. There were 544 males among the 1089 participants. Most of the flood survivors were keen to learn yoga and apart from the study yoga sessions were conducted for the others in the camp. Among 544 males, 65 participants met the other inclusion criteria. These were: (i) normal health, (ii) not on medication, (iii) readiness to be present for all assessments and to be assigned to either yoga or control group and (iv) no prior knowledge of yoga. Many participants had to be excluded as they had a diagnosed illness and were taking prescribed medication. A fifth factor which further limited the number (from 65 who met the four inclusion criteria mentioned above, to 28), was as follows. Considering that a month had elapsed after the floods, people were continuously being relocated to other temporary camps closer to the villages from which they came. Among the 65 flood survivors who met all our inclusion criteria 22 of them were told that they would not be transferred to another camp during the period of study. Hence the main factor which determined the sample size (which is small), was whether participants would be re-located to another camp during the study, as part of the attempts to restore normalcy and rehabilitate the survivors. The details have been given in a CONSORT diagram (see Figure 1).

The 22 participants constituted approximately 2 percent of the total sample of 1089 participants, but could be considered comparable to those of their age range and sex, in the larger sample. The 1089 participants who were in the camp at the time the present study was conducted as well as another 200 participants (making the total number 1289) were given the Screening Questionnaire for Disaster Mental Health (SQD) to determine the scores for post traumatic stress disorder and depression [12]. The SQD was administered to the participants 2 days before the present study as 2 days were required to screen the participants for the present study. The p (for PTSD) scores and d (for depression) scores of the 22 participants were not significantly different from the larger sample (n = 457) of males of a comparable age range [i.e., average p scores were 4.50 for the present group, and 4.37 for n = 457; average d scores were 3.35 for the present group, 3.19 for n = 457]. Apart from this, the group as a whole (as well as the 22 participants of the present study): (i) had all been directly affected by the floods, having lost their relatives, property, and friends, (ii) all of them had less than seven years of education, as after this most of them, particularly the males, had started learning job-related skills, and (iii) most of them were self-employed (i.e., owning small shops or working as farmers), while most of the females were house-wives. The sample size was not determined prior to the trial but was based on convenience. The study was approved by the Institution's Ethics Committee. The participants gave their signed consent to take part in the trial.

### Design

The study was a randomized controlled study. The 22 participants who were all males were randomized to two groups using a standard random number table [13]. The two groups (N = 11, each) were initially designated as Group 1 and Group 2 by a volunteer who had no role in the trial. The two groups were then designated as the 'yoga group' and 'the wait list control group' by the person from the research institution co-ordinating the activities at the camp, who picked up folded pieces of paper on which the name of the group was written. This person was not an experimenter, or a yoga teacher, and had no other part in the trial. The group mean ages ± S.D. were 32.1 ± 9.3 years for the yoga group and 30.8 ± 5.5 years for the control group. The yoga group practiced yoga for an hour daily for seven days and during this time the control group continued with the routine they were following in the camp. The yoga session was in the morning between 06:00 and 07:00 hours. All recordings were taken between 10:00 and 12:00 noon and 15:30 and 18:30 hours. The time of recording for each participant was kept constant for the initial and final assessment. The participants practiced yoga only during the one hour yoga session and they were instructed not to practice yoga at other times of the day. The control group did not practice yoga till the study was complete, when they were given the option to learn yoga if they wanted to. Further details about the two groups are given in Table 1. The trial was registered with the Clinical Trials Registry of India and assigned the registration number CTRI/2009/091/000285.

**Table 1:** Baseline characteristics of both groups

	Groups	
**Characteristics**	**Yoga (n = 11)**	**Control (n = 11)**

Age (years)	32.1 ± 9.3	30.8 ± 5.5

Years of education	6.4 ± 1.1	5.9 ± 2.0

Number who had themselves been directly affected by the floods	11	11

Number who had lost relatives or witnessed people dying	11	11

p > 0.05, comparing ages and years of education of the two groups using independent t tests

### Assessments

Assessments were done on the first day and eighth day of the study, a month after the floods, in October 2008. There were two categories of assessments; (i) autonomic and respiratory variables, and (ii) assessment of emotional responses using visual analog scales (VAS). The main outcome measures were the symptoms of emotional distress based on visual analog sales and the secondary outcome measures were the heart rate variability and breath rate, recorded using a polygraph. Visual analog scales were chosen rather than validated questionnaires, even though it was considered a limitation of an earlier published study [8] since it was easier to get accurate responses to the VAS from the participants, all of whom had less than 7 years of education.

All assessments were performed in the camp in a tent set aside for testing. One of the experimenters had previously administered the VAS used here to tsunami survivors [8] and had seven years of experience in using a polygraph to record heart rate variability and respiration.

#### (i) Autonomic and respiratory variables

Heart rate variability was assessed using a 4 channel digital polygraph (Recorders & Medicare, Chandigarh, India). The EKG was recorded using Ag/AgCl pre-gelled electrodes (Tyco Healthcare, Deutschland, Germany) and recording was made using a Standard Limb Lead I configuration. Data were acquired at the sampling rate of 1024 Hz and were analyzed offline. Noise free data were used for analysis. The data were analyzed using software developed by the Biomedical Signal Analysis Group, University of Kuopio, Finland [14]. The heart rate variability was recorded for five minutes for each participant. During the recording participants were asked to lie down, supine. The respiration was recorded using a volumetric pressure transducer fixed around the trunk about 8 cm below the lower costal margin as the subject was lying on a bed.

#### (ii) Assessment of emotional responses using visual analog scale (VAS)

For the participants of the present study (n = 22) it was decided to use visual analog scales (i) as it was easier to get accurate responses, and (ii) comparisons were planned with an earlier study which used the same VAS in tsunami survivors following a week of yoga [8]. However, it is recognized that using visual analog scales instead of validated questionnaires is a limitation of the study.

Visual analog scales (VAS) were designed for participants to self-rate their (i) fear, (ii) anxiety, (iii) disturbed sleep, and (iv) sadness as these are indicators of emotional distress commonly reported by disaster survivors [15]. Each analog scale was a 10 centimeter long doubly anchored scale, with one end (score = 10) of the scale indicating the highest intensity of a feeling of a symptom of PTSD, while the other end (score = 0) indicated the lowest intensity of feeling for the same symptom. There was a separate scale for each of the four symptoms. Participants were instructed to place a vertical mark on the horizontal line to indicate the level of their feelings. For each individual the score for a particular symptom was obtained by measuring the distance in millimeters from the end of the line where the score was '0' upto the mark made by the subjects. All the analog scales were scored in one direction (i.e., with '0' on the left), to make it easier to explain the method to the participants. Hence for each of the four symptoms (i.e., fear, anxiety, disturbed sleep, and sadness), separate scores were obtained as millimeters for each of the four VAS.

### Intervention

The yoga group practiced yoga for an hour daily for seven days and during this time the control group continued with the routine they were following in the camp. The yoga session was in the morning between 06:00 and 07:00 hours. The yoga class included loosening exercises (*sithilikarana vyayama*) for ten minutes, physical postures (*asanas*) for twenty minutes and breathing techniques (*pranayamas*) for twenty five minutes. These practices were followed by five minutes of guided relaxation in *shavasana *(corpse pose).

Loosening exercises (*sithilikarana vyayama*) are a set of techniques which involve repetitive movements of all joints from the toes up to the neck to increase mobility and to prepare for the practice of yoga postures.

The following yoga postures were practiced: standing posture (*tadasana*), lateral arc posture (*ardhakaticakrasana*), hand-to-foot posture (*padahastasana*), half wheel posture (*ardhacakrasana*), back-stretching posture (*paschimottanasana*), half lotus posture (*ardha padmasana*), moon posture (*sasankasana*), crocodile posture (*makrasana*), cobra posture (*bhujangasana*), locust posture (*shalabhasana*), shoulder stand posture (*sarvangasana*), and fish posture (*matsyasana*).

The breathing techniques included high frequency yoga cleansing breathing (*kapalabhati*), alternate nostril yoga breathing (*anulom-vilom pranayama*), exhalation while making a humming sound like a bumble bee (*brahmari pranayama*) and exhalation with chanting of a syllable, OM (*udgheeth pranayama*). The breath rate for the high frequency yoga cleansing breathing (*kapalabhati*) was approximately 60 breaths per minute. For alternate nostril breathing (*anulom-vilom pranayama*) the breath rate was approximately 12 breaths per minute, whereas for the breathing practices involving exhalation with a sound (e.g., *brahmari *and *udgheeth pranayamas*), the breath rate was lower, approximately 8 breaths per minute. In the present study the breath rate was not recorded during the practice of different yoga breathing techniques. The breath rates mentioned here are based on our unpublished data recorded in normal volunteers who were also novices to yoga and learned the techniques in comparable time.

This yoga program has been called Patanjali yoga as it is based on the teachings of Patanjali (*circa *900 B.C.). It is taught by Swami Ramdev. None of the participants reported any adverse effects of the program.

### Data extraction

The HRV power spectrum was obtained using Fast Fourier Transform (FFT) analysis. The energy in the HRV series in specific frequency bands was studied viz., very low frequency (VLF) band (0.0-0.04 Hz), low frequency (LF) band (0.5-0.15 Hz), high frequency (HF) band (0.15-0.50 Hz) and the LF/HF ratio. The very low frequency, low frequency and high frequency band values were expressed as normalized units [16]. In addition to the frequency domain analysis, time domain analysis was also done. The following components of time domain HRV were analyzed, viz., the pNN50, the proportion of R-R intervals having a difference more than 50 msec and NINN which is a triangular index, giving the integral of the density distribution (i.e., the number of all NN intervals plotted in a histogram) divided by the maximum of the density distribution. Emotional impact in terms of fear, anxiety, disturbed sleep and sadness were calculated by measuring the distance in millimeters from the left of the analog scale (where the left end of the scale corresponded to '0' and the right end to '10'). All the analog scales were scored in one direction to make it easier to explain the method to the participants.

All assessments (i.e., the four VAS, heart rate variability, and breath rate) were blind scored by an investigator who did not know to which group a participant belonged. The success of blinding was not evaluated. The yoga instructor led the yoga sessions but had no part in randomizing the participants to two groups, assigning the two groups to an intervention, or in scoring the data.

### Data analysis

Data were analyzed using SPSS (Version 16.0). A repeated measures ANOVA (with Groups as the Between Subjects factor and Assessments as the Within Subjects factor) was performed. There were no significant differences between Groups or pre-post Assessments, and the interaction between Groups and Assessments was also not significant, hence *post-hoc *analyses were not attempted as they would have had no validity [17]. Pre-post comparisons were made using a t-test for paired data. The pre values of the two groups were compared with independent t-tests.

## Results

### Repeated measures analysis of variance

Repeated measures analyses of variance were performed. There were no significant differences between Groups, Assessments, or Interaction (Groups × Assessments) for the four VAS end-points, heart rate variability, or breath rate (p > 0.05, for all comparisons).

With no significant differences between Groups, Assessments or Interaction between the two, follow-up *post-hoc *tests were not done as they would have no validity [17]. Instead, t-tests were performed, with Bonferroni correction as described below.

### Multiple t-tests for paired and unpaired data

#### (i) Autonomic and respiratory variables

No significant changes were observed in the heart rate variability (HRV) and breath rate of both groups. The group mean values ± SD are given in Table 2.

**Table 2:** Heart rate variability values and breath rate in yoga and control groups. Values are group mean (SD)

	Yoga (n = 11)		Control (n = 11)	
**Variables**	**Pre**	**Post**	**Pre**	**Post**

LF (n.u.)	56.54 (17.62)	55.76 (24.47)	53.93 (23.67)	50.24 (16.12)

Hf (n.u.)	43.40 (17.67)	44.19 (24.46)	46.07 (23.67)	49.73 (16.24)

LF/HF	1.23 (0.75)	1.77 (1.46)	1.25 (0.83)	1.42 (0.87)

NN50	54.13 (56.79)	58.13 (87.41)	30.08 (39.42)	60.08 (60.19)

pNN50	13.33 (12.78)	21.16 (29.20)	7.56 (10.32)	16.79 (16.97)

TINN	390.00 (504.28)	695.63 (676.35)	430.42 (399.60)	250.42 (177.99)

Breath rate (cpm)*	17.86 (4.24)	16.63 (4.11)	18.02 (2.66)	18.78 (2.38)

*cpm = cycles per minute

#### (ii) Visual analog scales

There was a significant reduction in sadness in the yoga group (p < 0.05 based on a pre-post comparison with a paired t test; here p = 0.021; and after Bonferroni correction for 2 paired comparisons, p = 0.042) and a significant increase in anxiety in the control group (p < 0.05 based on a pre-post comparison with a paired t test; here p = 0.023; and after Bonferroni correction for 2 paired comparisons, p = 0.046).

There were no significant differences between the pre values of the two groups (i.e., p > 0.05 comparing pre values of the two group using independent t-tests). The group mean values ± SD are given in Table 3.

**Table 3:** Visual analog scale responses in both groups. Values are group mean (SD)

	Yoga (n = 11)		Control (n = 11)	
**Variables**	**Pre**	**Post**	**Pre**	**Post**

Fear (mm)	3.64 (2.43)	2.37 (2.71)	3.19 (2.65)	4.90 (3.59)

Anxiety (mm)	5.72 (3.19)	4.49 (2.64)	4.76 (2.69)	4.88 (3.15)*

Sadness (mm)	7.12 (3.21)	5.98 (3.58)*	6.25 (2.75)	5.07 (2.89)

Disturbed sleep (mm)	2.59 (3.47)	3.04 (3.44)	2.26 (3.29)	4.03 (3.91)

*p < 0.05, comparing pre and post values with a t-test for paired data; p > 0.05 comparing pre values of the two group using independent t-tests

## Discussion

In the present randomized controlled trial, a month after the floods in the north Indian state of Bihar, the effects of a week long yoga intervention were studied in the survivors. Following a week of yoga practice, survivors showed a significant decrease in self-rated sadness while the non-yoga control group showed an increase in self-rated anxiety. Neither group showed changes in heart rate variability or in breath rate.

Previously a one week yoga program reduced self-rated fear, anxiety, sadness and disturbed sleep, as well as decreased heart and breath rates in tsunami survivors a month after the calamity [8]. The yoga program was for 60 minutes each day which was, the same duration as the present study. In the case of the tsunami survivors the yoga program consisted of yoga postures (*asanas*, with 16 postures in 20 minutes), loosening exercises (for 10 minutes), yoga voluntarily regulated breathing (for 15 minutes, with four practices), and guided relaxation (for 15 minutes). In the present study the yoga program had the same components but with slight variations, as mentioned below. The program consisted of yoga postures (*asanas *with 12 postures in 20 minutes), loosening exercises for 10 minutes, yoga voluntarily regulated breathing (for 25 minutes with 3 practices) and guided relaxation for 5 minutes. It is unlikely that the difference (mainly an increased time spent on voluntarily regulated breathing and less time spent in guided relaxation) accounted for the differences in results. One of the main differences is that the present study had a considerably smaller sample size (n = 11), compared to the earlier study [8], where the sample size was 47. The populations also differed in that the tsunami survivors were exposed to the trauma for the first time, whereas the participants in the present study had previous exposures to the trauma. The small sample size is a serious limitation of the study and was mainly due to the fact that participants were continuously being re-located to other camps, elsewhere.

A yoga breath intervention which included SKY, was found to relieve psychological distress in survivors of the 2004 South-East Asia tsunami [18]. In this non-randomized study, 183 tsunami survivors with scores of 50 or more on the post-traumatic checklist-17 (PCL-17) were assigned to three groups. The three groups were yoga breath intervention, yoga breath intervention followed by 3-8 hours of trauma reduction exposure technique or a wait-list control group. This assignment was for participants within different camps and hence was camp-based. Assessments for post-traumatic stress disorder and depression were performed at 6, 12 and 24 weeks. Scores for post traumatic stress disorder (based on the PCL-17) decreased in the group assigned to yoga breath and in the group with yoga breath with exposure, though it was more in the former group. This study [18], unlike the present study had a large sample, a long duration of follow-up, and used validated instruments. Nonetheless, both reports suggest the benefits of a yoga program which emphasized breathing techniques for PTSD.

Healthy people, without any psychological illness showed increased levels of optimism and reduced levels of depression after practicing SK&P for six weeks [19]. Yoga breathing practices were also found to benefit African-American and European women who had been abused and gave testimony about intimate partner violence [20]. Learning yoga breathing techniques alone as well as in combination with giving testimony reduced feelings of depression. It was speculated that teaching women to calm their minds by focusing on their breath helped them to take control of their bodies and their lives.

The non-yoga control group in the present study showed a significant increase in self-rated anxiety. A combination of yoga postures and yoga breathing (similar to the practices used here) reduced state anxiety in normal volunteers [21]. Also, in another study, a combination of yoga breathing exercises and meditation reduced symptoms of anxiety and depression, while increasing feelings of wellbeing, compared to the control group [6]. The mechanism by which yoga breathing may be reducing anxiety and increasing feelings of wellbeing is not known. However slow and deep breathing is known to increase the parasympathetic tone and is associated with a calm mental state [22]. This is of relevance as some of the yoga breathing techniques which formed part of the present study included slow and deep breathing. Apart from slow breathing, the present program also included rapid breathing techniques, which have been shown to be followed by periods of slow electroencephalogram (EEG) frequencies and subjectively rated calmness [23]. In fact, the close connection between emotional states and breathing has been demonstrated since six basic emotions have characteristically different sets of breathing patterns [24]. The six basic emotions are joy-laughter, sadness-crying, fear-anxiety, anger, erotic love and tenderness. The close connection between yoga breathing and emotions including anxiety, suggests that yoga practice may have prevented the increased anxiety seen in the control group.

The increase in self-rated anxiety in the control group needs further explanation. This is particularly the case as previous studies have not demonstrated an increase in anxiety in untreated trauma survivors [25], or have shown an actual reduction in those treated with anti-anxiety medication [26] or group debriefing as an immediate effect of the session [27]. A possible reason for the increased anxiety in the control group may be the fact that the survivors were not receiving the kind of assistance they needed with the necessary speed. Social scientists reported that in the initial phase the administration, civil society groups and the media kept seeing the disaster as an 'annual flood' which was nothing new for that part of the country [28]. All possible sources of aid failed to recognize the magnitude of the calamity. Hence given their dissatisfaction with the way in which relief was being provided the control group may have shown an increase in anxiety, which possibly was prevented from happening in the yoga group by the practice of yoga. Apart from this, the fact that the control group did not have interaction with an instructor could have contributed to their increased anxiety levels, as additional care and attention given by an instructor or healer are known to have psychological benefits [29]. However the small sample size and consequently the use of less rigorous statistical analysis prevent definite conclusions from being made.

The absence of change in fear and sleep disturbances in the present study may be related to the fact that the intervention focused more on voluntarily regulated yoga breathing than on yoga postures (*asanas*), as voluntarily regulated yoga breathing constituted 50 percent of the total time spent in yoga practice. The practice of yoga postures along with guided relaxation in the day time was associated with an increase in slow wave sleep and a decrease in REM sleep on the subsequent night [30]. In this study [30], yoga postures were believed to act as a form of mild exercise and exercise is known to promote sleep. The effect of yoga voluntarily regulated breathing on sleep has not been studied. The lack of effect on sleep disturbances may hence be related to the fact that at least half the time was spent in yoga voluntarily regulated breathing rather than yoga postures, through the latter are known to have a favorable effect on sleep.

In attempting to understand the contribution of individual practices to the effects seen, previous studies have shown that the practice of yoga postures interspersed with relaxation while supine reduced sympathetic nervous activity more than a comparable period of supine rest alone [31]. Also, the same combination of postures and supine rest delayed the latencies of certain evoked potential components which are generated in the cerebral cortex [32]. Apart from this, an hour of practicing yoga postures increased the levels of the inhibitory neurotransmitter gamma-aminobutyric acid (GABA) compared to an equal duration of time spent reading [33]. The individual effects of separate *asanas *have not been worked out. Apart from yoga postures, loosening exercises (*sithilikarana vyayama*) were shown to increase flexibility and reduce musculoskeletal discomfort in professional computer users [34]. The effects of yoga breathing practices have been assessed more individually. High frequency yoga breathing (*kapalbhati*) has been shown to increase the low frequency power of heart rate variability suggesting an increase in sympathetic nervous system activity [35]. In contrast alternate nostril yoga breathing (*anulom-vilom pranayam *[36] reduced the systolic, diastolic, and mean pressure values suggestive of lower sympathetic nervous system activity. Hence there may have been no overall effect of yoga voluntarily regulated breathing (*pranayama*) on the sympathetic nervous system activity in participants, which may have been the reason why there was no change in the heart rate variability. Also at present it is not possible to specify which specific yoga practice may be responsible for a particular effect (e.g., reducing sadness or preventing the yoga practitioners from feeling more anxious than they did at the beginning of the week, which was seen in the non yoga/control group).

In the present study there was also no change in the level of fear based on a VAS. Among various levels of fear, possibly the most extreme is the fear of death. A one year longitudinal study of two groups [yoga, (emphasizing Buddhist principles of mindfulness, compassion and equanimity) and a control group], showed that the yoga group had decreased fear of death at the end of the year [37]. In the present study there was no attempt to add philosophical aspects of yoga, which include discussions of fear and how to deal with it [2]. This may be considered a limitation of the intervention. However, the philosophical principles of yoga are drawn from ancient texts, often associated with Hindu spiritual beliefs. Since the flood survivors belonged to different faiths some of these principles may not have been acceptable to them and hence were not added.

The absence of change in the heart rate variability may be related to the yoga program and the yoga breathing techniques in particular. The breath rate and heart variability (HRV) are closely related. Respiratory sinus arrhythmia (RSA) is a commonly employed non-invasive measure of cardiac vagal control [38]. Respiratory variables such as tidal volume and breath rate have been shown to change with no change in tonic vagal activity. Hence, concurrent monitoring of respiration and physical activity are considered likely to enhance HRV accuracy to predict autonomic control. This is supported by acute increases in low frequency and total spectrum HRV and in vagal baroreflex gain, which is corrected by slow breathing periods with biofeedback [39]. It was earlier shown that biofeedback training increased the amplitude of heart rate oscillations at approximately 0.1 Hz [40]. To achieve this, breathing is slowed to a point at which resonance occurs between respiratory-induced oscillations and oscillations which naturally occur at this rate. Previously, studies on the effects of specific yoga practices examined whether a decrease in breath rate could have influenced the HRV where an increase in LF power would be related to slower breathing rather than autonomic activity [30]. In contrast, fast breathing practices (*kapalabhati*) have been associated with increased sympathetic nervous system activity [41], while slower breathing practices (*anulom-vilom pranayama*) have been associated with reduced sympathetic nervous system activity [36]. In the present study the intervention consisted of both fast and slow breathing practices. As a result the heart rate variability may not have changed, or reduced as much as in cases where the intervention consisted of slow breathing practices alone. The end result may have been no change in the heart rate variability.

In addition to yoga, other mind-body interventions have been found to be useful for trauma victims. One hundred and thirty-nine high school students in Kosovo participated in three separate programs which included several mind-body interventions spaced two months apart [42]. The interventions included meditation, biofeedback, autogenic training, guided imagery, movement, and breathing techniques. The adolescents showed significantly lower symptoms of post traumatic stress based on the Posttraumatic Stress Reaction Index, compared to the initial values. In another study refugees and survivors of torture appeared to respond positively to the practice of *qi gong *and *t'aichi*, based on observations made on four refugee survivors [43].

However, it is understood that assessing the short term impact of an intervention may not give adequate information about its' efficacy [44]. When road traffic accident victims received psychological debriefing, the outcome assessed at three years showed that the intervention group had a significantly worse outcome in terms of general psychiatric symptoms, as well as other problems. In another report also, individual single-session psychological debriefing was shown to aggravate symptoms of PTSD at six weeks in those participants in the intervention group who had high baseline hyper arousal scores [45]. Hence in the present study a long term follow-up would have given useful information and not having such a follow-up limits interpreting and using the findings.

Apart from the lack of follow-up, the other serious limitation was a small effect size. The small effect size may be due to at least two factors. The most important reason is the small sample size, which is recognized as a serious limiting factor of the study. The other factor could be that the follow-up period was one week (as mentioned above). After a longer duration of yoga practice a greater magnitude of change may have occurred. There were two reasons why the duration of the follow-up period was kept as one week. The first reason was a practical reason. The flood survivors were continuously being relocated to other camps closer to the villages from which they came. The second reason was that in an earlier study a one week yoga intervention (which had almost the same yoga techniques but for slightly different durations) had been used, a month after the event, for tsunami survivors [8]. The symptoms of distress in the tsunami survivors were assessed using the same visual analog scales as those used in the present study. It was intended to compare the two groups which had common features (e.g., being given a week of yoga practice a month after a natural disaster) as well as differences (e.g., the yoga program was different, the present study had a non-yoga group for comparison, in the present study the sample size was smaller, and the flood survivors regularly faced the trauma of the floods, as this happens every year, though the magnitude of the problem differs).

Despite these limitations the present findings suggest that a week long yoga intervention can reduce self-rated sadness and may prevent an increase in anxiety in survivors of floods. This may be particularly important in developing countries and in the case of survivors of recurrent disasters, where the survivors would know the outcome and hence may have specific apprehensions, for example, that aid may be delayed or inadequate, based on their earlier experiences.

## Conclusions

It was observed that following the seven days of yoga practice there was a reduction in sadness based on a pre-post comparison using a t test for paired data while the non-yoga group had an increase in anxiety, also based on a pre-post comparison using a t test for paired data.

## List of abbreviations

PTSD: Post traumatic stress disorder; VAS: Visual analog scale; EKG: Electrocardiogram; HRV: Heart rate variability; FFT: Fast fourier transform; VLF: Very low frequency; LF: Low frequency; HF: High frequency; SPSS: Statistical package for social sciences; SK&P: *Sudarshan kriya *and related practices; EEG: Electroencephalogram.

## Competing interests

The authors declare that they have no competing interests.

## Authors' contributions

ST designed the study, interpreted the data and wrote the manuscript. NS and MJ collected the data, performed the statistical analyses and helped to compile the manuscript. AB gave advice about the intervention and collected funds for the project. All authors have read and approved the final manuscript.

## Pre-publication history

The pre-publication history for this paper can be accessed here:

http://www.biomedcentral.com/1471-244X/10/18/prepub
